# Expression of fibronectin in esophageal squamous cell carcinoma and its role in migration

**DOI:** 10.1186/s12885-018-4850-3

**Published:** 2018-10-12

**Authors:** Jiefei Xiao, Weilin Yang, Bo Xu, Haoshuai Zhu, Jianyong Zou, Chunhua Su, Jian Rong, Tao Wang, Zhenguang Chen

**Affiliations:** 1 Department of Extracorporeal Circulation, the First Affiliated Hospital, Sun Yat-sen University, Guangzhou, 510080 Guangdong China; 2 Key Laboratory on Assisted Circulation, Ministry of Health, Guangzhou, 510080 China; 3 Department of Cardiothoracic Surgery of East Division, the First Affiliated Hospital, Sun Yat-sen University, No. 58, Zhongshan Road II, Guangzhou, 510080 Guangdong China; 4 Department of Thoracic Surgery, the First Affiliated Hospital, Sun Yat-sen University, No. 58, Zhongshan Road II, Guangzhou, 510080 Guangdong China; 5 Lung Cancer Research Center of Sun Yat-sen University, Guangzhou, 510080 Guangdong China; 6 Center for Stem Cell Biology and Tissue Engineering, Sun Yat-sen University, Key Laboratory for Stem Cells and Tissue Engineering, Ministry of Education, Guangzhou, 510080 Guangdong China; 7 Department of Biochemistry, Zhongshan Medical School, Sun Yat-sen University, Guangzhou, 510080 Guangdong China

**Keywords:** Esophageal squamous cell carcinoma (ESCC), Fibronectin (FN), Migration, Prognosis, Tumor microenvironment

## Abstract

**Background:**

Fibronectin (FN) is a high-molecular-weight glycoprotein component of the extracellular matrix involved in cell adhesion, migration, metastasis, proliferation and differentiation, as well as embryogenesis, wound healing, and blood coagulation. Considerable recent research has established that tumor expression of FN is closely associated with tumor formation and development as well as disease prognosis. However, the mechanisms underlying this relationship have remained unclear. The aim of this study was to investigate FN protein expression in esophageal squamous cell carcinoma (ESCC) and determine its potential prognostic relevance, while also elucidating the source and function of FN.

**Methods:**

We conducted immunohistochemical analyses of protein expression in primary tumors of ESCC patients and analyzed their association with standard prognostic parameters and clinical outcomes. Expression of FN in two ESCC cell lines (Eca-109 and TE-1) was also examined by RT-PCR, immunofluorescence, and ELISA. ESCC cells were cultured in a microenvironment containing a high FN content, and changes in their morphology and migration ability were assessed by microscopy, wound-healing assays, and Transwell assays.

**Results:**

FN expression in ESCC specimens was mainly detected in the tumor stroma, with very little FN detected in tumor cells. Stromal FN content in ESCC specimens was associated with lymphatic metastasis (*P* = 0.032) and prognosis. In this latter context, patients with high tumor stromal expression of FN showed worse overall survival (*P* = 0.002) and progression-free survival (*P* < 0.001) than those with low expression of FN. Interestingly, FN expression and secretion in ESCC cell lines (Eca-109 and TE-1) was found to be low, but these cells adopted a more migratory phenotype when cultured in vitro in a microenvironment containing high levels of FN.

**Conclusions:**

High FN expression in the stroma of ESCC tumors is closely associated with poor prognosis of patients. High stromal FN content facilitates tumor cell metastasis by promoting morphological changes and improving the motility and migratory ability of ESCC cells.

## Background

Esophageal cancer is the sixth-leading cause of cancer-related mortality and the eighth-most common cancer worldwide [1]. In the United States alone, 16,940 new cases and 15,690 deaths of esophageal cancer occurred between January and October in 2017 [2]. There are two main pathological types of esophageal cancer: squamous cell carcinoma and adenocarcinoma. Esophageal squamous cell carcinoma (ESCC) is a major histological subtype of esophageal carcinoma that is frequently diagnosed in East Asian countries, especially in China [3]. The current standard treatment for esophageal cancer is surgery in conjunction with treatments based on chemotherapy and radiotherapy, among others. However, despite improvements in surgery and chemo/radiotherapy, the prognosis for ESCC patients remains poor. One of the major reasons for treatment failure is tumor recurrence or metastasis. Thus, studies of the mechanism and improvements in diagnosis and therapy are important for enhancing the 5-year survival and quality of life of esophageal cancer patients.

Fibronectin (FN), a high-molecular-weight glycoprotein component of the extracellular matrix, exists in three forms: cellular FN, plasma FN and fetal FN [4]. FN consists of two subunits with a molecular weight of 220–225 kDa linked via a disulfide bond. Each subunit contains several ligand-binding domains, allowing FN to mediate activation of a series of signal transduction pathways and thereby regulate cellular processes such as adhesion, migration, proliferation and differentiation, among others [5].

Expression of FN in several types of cancer, including breast cancer, lung cancer, thyroid cancer, oral squamous cell carcinoma and esophageal cancer, among others, has been reported based on immunohistochemical analyses [6–11]. It has further been demonstrated that that high expression of vimentin and FN is associated with advanced stage and poor prognosis in ESCC [12].

In this study, we performed immunohistochemical analyses of ESCC tissue samples and correlated FN expression with clinicopathologic features and patient survival so as to clarify the prognostic significance of FN expression in ESCC. We also assessed FN expression in ESCC cell lines, and monitored changes in the morphology and migration ability of ESCC cells cultured in a microenvironment containing a high FN content. Collectively, our findings suggest a role for stromal FN in facilitating the escape and metastasis of ESCC cells.

## Methods

### Case selection and cell lines

For this study, we collected 68 cases of ESCC that had undergone surgical resection at the First Affiliated Hospital of Sun Yat-sen University between September 2010 and March 2012. All cases were pathologically confirmed to be ESCC. The age and gender of the patients, histologic grade, pathologic tumor stage (pT), pathologic lymph node stage (pN), TNM stage, surgery type, and follow-up information were collected from the medical records of patients. Survival information was attained through telephone contact or outpatient service. The use of human materials was approved by the Medical Ethical Committee of The First Affiliated Hospital, Sun Yat-sen University (Full name of the board/committee: The Medical Ethical Committee of The First Affiliated Hospital, Sun Yat-sen University). We confirm that written informed consent from the donor or the next of kin was obtained for use of this sample in research.

The two ESCC cell lines, Eca-109 (TCHu 69) and TE-1 (TCHu 89), were obtained from the Cell Bank of the Chinese Academy of Science (Shanghai, China). Mesenchymal stem cells (MSCs) were derived from a primary human MSC at the Center for Stem Cell Biology and Tissue Engineering, Sun Yat-Sen University. The center recruited healthy donor for bone marrow MSCs from the present study of mesenchymal stem cells for the treatment of graft-versus-host diseases and the remaining MSCs were selected for our experimental study [13–15].

### The primary antibody

The primary antibody used in this study was antifibronectin rabbit monoclonal antibody (ab32419, Abcam, Cambridge, UK). Indeed, fibronectin gene encodes many different isoforms of fibronectin protein. The antifibronectin rabbit monoclonal antibody we used in our experiment is fit for 17 isoforms of fibronectin protein according to its product information and it would cover most of the isoforms. The antibody is widely used according to some similar experiments [16–19].

### Immunohistochemical staining and evaluation

The primary antibody used in this study was anti-FN (ab32419, diluted 1:200; Abcam, Cambridge, UK). Immunohistochemical staining was carried out using the streptavidin-peroxidase method. FN staining was scored on a 1-to-4 scale, as follows: 1, < 25% staining; 2, 25% to 50% staining; 3, 50% to 75% staining; 4, > 75% staining. Ten photographs of tumor tissue were taken randomly; each was graded and the final score was presented as the average value. The median value of FN was used to classify samples as high-FN expression (above the median) or low-FN expression (below the median).

### Immunofluorescence

Eca-109 and TE-1 cell lines were cultured in 24-well plates to a density of approximately 70–80%. After discarding the medium and washing three times with 0.01 M phosphate-buffered saline (PBS), cells were fixed with 4% paraformaldehyde. Fixed cells were first incubated overnight at 4 °C with anti-FN primary antibody (ab32419, diluted 1:200; Abcam) and then with fluorescence-conjugated anti-IgG secondary antibody (Life Technologies) in the dark. Nuclei were counterstained with 4′,6-diamidino-2-phenylindole (DAPI; Roche, Switzerland), after which cells were observed and photographed under an inverted fluorescence microscope.

### Reverse transcription-polymerase chain reaction (RT-PCR)

Total RNA was extracted using an RNA kit (Qiagen, Germany), and reverse transcription was performed using a reverse transcription kit (Thermo-Fisher Scientific) according to the manufacturers’ instructions. Real-time quantitative reverse transcription-PCR (qRT-PCR) was performed on a Step-One Plus (Applied Biosystems, Foster City, CA, USA) using SYBR Green reagents (Roche, Switzerland) and the following primer pairs: FN, 5’-ACC TCG GTG TTG TAA GGT GG-3′ (forward) and 5’-CCA TAA AGG GCA ACC AAG AG-3′ (reverse); and glyceraldehyde-3-phosphate dehydrogenase (GAPDH), 5′-GAA GGT GAA GGT CGG AGT C-3′ (forward) and 5′-GAA GAT GGT GAT GGG ATT TC-3′ (reverse). The relative mRNA expression level of the target gene was normalized to that of GAPDH, and expressed as fold-change relative to Non-SP cells.

### Enzyme-linked immunosorbent assay (ELISA)

The levels of FNin conditioned medium of ESCC cell lines (Eca-109, TE-1) and MSCs were measured using the ELISA kit (BMS2028/BM2028TEN; eBioscience) according to the manufacturer’s instructions. Each sample was analyzed in triplicate. After development with a chromogen-substrate solution, the reaction was terminated by adding 100 μl of stop solution. Optical density values were read at 450 nm, and the concentrations were automatically calculated according to the standard curve.

### Production of MSC-conditioned medium concentrates

MSCs were grown in a culture bottle to maximum density, after which the medium was replaced with serum-free, DMEM/low carbohydrate medium without penicillin-streptomycin (Gibco). After further culturing MSCs for 3 days, the culture medium was collected, filtered, concentrated, and stored at − 80 °C.

### Morphological assay

Eca-109 and TE-1 cells were first cultured overnight in 6-well plates (~ 0.8 × 10^5^ cells/well), after which the medium was removed and replaced with 2 ml of fresh serum-containing DMEM/low carbohydrate medium. Concentrated MSC-conditioned medium (500 μl) was added to cells in the experimental group, and an equal volume of serum-free DMEM/low carbohydrate culture solution without penicillin-streptomycin was added to cells in the control group. Cells were cultured at 37 °C in a humidified 5% CO_2_ environment, and monitored and photographed every 6 h.

### Wound-healing assay

Mark lines behind the 6 well plates, 5 lines one plate. Inoculate about 5 × 10^5^ cells in every plate, and cultivate overnight. The cells were cultured in serum-containing medium for 24 h, and then gaps of 1 mm open spaces were generated manually by scratching the monolayer of cell culture., after which the medium was removed and washed three times use PBS to remove the cells healled down, and replaced with 1 ml of fresh serum-containing DMEM/low carbohydrate medium in every plate. The “healing” effect was monitored microscopically periodically and taken photographs as the cells migrate to cover the blank surface.

### Transwell assays

Migration assays were conducted using Transwell inserts with 8-μm pores (Millipore) according to the manufacturer’s instructions. Briefly, after washing ESCC cells twice in serum-free medium, 2 × 10^4^ cells were resuspended in 200 μl of fresh serum-free medium and seeded into the upper chamber of a 24-well plate. The lower chamber contained 200 μl serum-containing DMEM medium, to which was added 300 μl of concentrated MSC-conditioned medium (experimental group) or 300 μl serum-free medium (control group). The cells were allowed to migrate for 12 h at 37 °C, after which the chambers were washed with PBS, and cells on the lower surface of the chamber were stained with 0.1% crystal violet stain solution and counted in four different random fields at × 40 magnification under an electron microscope. Each experiment was performed at least three times.

### Statistical analysis

All calculations were done using SPSS V13.0 software. Spearman’s coefficient of correlation, Chi-squared tests and Mann-Whitney tests were used, as appropriate. A multivariate model was used to evaluate statistical associations among variables, and a Cox regression model was used to relate potential prognostic factors with survival. *P*-values < 0.05 were considered statistically significant.

## Results

### FN expression and clinical significance in ESCC

**Fig. 1:**
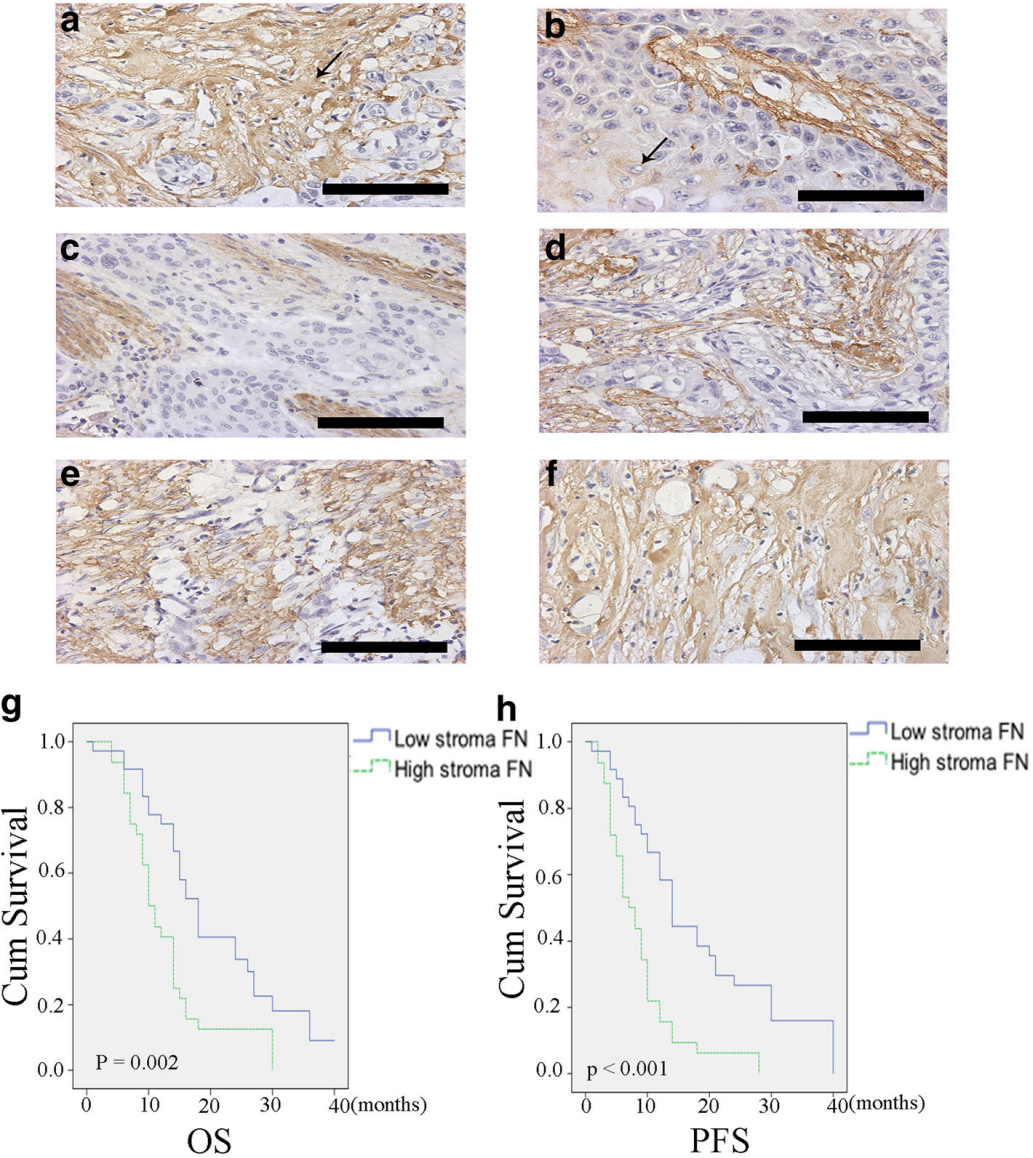
FN expression and clinical significance in ESCC patients. **a**, **b** Immunohistochemical analyses showing that FN is mainly expressed in the tumor stroma (**a**), with less frequent expression in tumor cells (**b**). Magnification, × 400. (**c–f**). Immunohistochemical grading standards, 1–4, corresponding to stained areas of < 25% (**c**), 25–50% (**d**), 50–75% (**e**), and > 75% (**f**), respectively. Magnification, × 400. (**g, h**) Kaplan-Meier survival curves for patients with ESCC, showing poorer OS (**g**; *P* = 0.002) and PFS (**h**; *P* < 0.001) for patients with high stromal FN expression compared to those with low stromal FN expression. Scale bar = 100 μm

We collected and analyzed clinical details and pathological characteristics of 68 patients (Table 1). FN expression was divided into two classes: high-FN and low-FN, using the median expression level as the cutoff point. Stromal FN content was high in 32 of 68 cases (47.1%) and low content in 36 of 68 cases (52.9%). Stromal FN expression in ESCC was significantly correlated with pN (*P* = 0.032); 17 of 45 N0 patients (37.8%) had high stromal FN content, whereas 15 of 23 N1–3 patients (65.2%) high stromal FN content. There were no significant differences in stromal FN expression according to gender, age, tumor differentiation, pT status or TNM stage. Immunohistochemical analyses showed that FN was mainly expressed in the stroma of the tumor, with much lower expression in tumor cells (Fig. 1). Notably, Kaplan-Meier survival curve analyses showed that higher stromal expression of FN was associated with poorer overall survival (OS) and progression-free survival (PFS) in patients with ESCC (OS, *P* = 0.002; PFS, *P* < 0.001). To investigate stromal FN expression as risk factor for prognosis, we performed univariate and multivariate analyses of OS and PFS in patients with ESCC (Tables 2 and 3). Lymph node metastasis (hazards ratio [HR], 2.151; 95% confidence interval [CI], 1.246–3.715; *P* = 0.006), TNM stage (HR, 1.875; 95% CI, 1.087–3.235; *P* = 0.024), and stromal FN content (HR, 2.163; 95% CI, 1.274–3.671; *P* = 0.004) showed a significant association with OS in univariate analyses, whereas lymph node metastasis (HR, 2.188; 95% CI, 1.281–3.736; *P* = 0.004) and stromal FN content (HR, 2.813; 95% CI, 1.648–4.804; *P* < 0.001) showed a significant association with PFS. Stromal FN expression (OS: HR, 2.022; 95% CI, 1.169–3.498; *P* = 0.012; PFS: HR, 2.498; 95% CI, 1.444–4.319; *P* = 0.001) and lymph node metastasis (OS: HR, 2.158; 95% CI, 1.207–3.857; *P* = 0.009; PFS: HR, 1.783 95% CI, 1.031–3.082; *P* = 0.039) were independent prognostic factors for OS and PFS in patients with ESCC.

**Table 1:** ESCC patient characteristics according to tumor stromal FN expression

Characteristics	No. of patients	High stromal FN	Low stromal FN	*P*-value
Gender				0.490
Male	55	27 (49.1%)	28 (51.0%)	
Female	13	5 (38.5%)	8 (61.5%)	
Age (years)				0.491
≤ 60	37	16 (43.2%)	21 (56.8%)	
> 60	31	16 (51.6%)	15 (48.4%)	
Tumor differentiation				0.881
Poor	24	11 (45.8%)	13 (54.2%)	
Well+Moderate	44	21 (47.7%)	23 (52.3%)	
pT status				0.228
T1 + T2	15	5 (33.3%)	10 (66.7%)	
T3	53	27(50.9%)	26 (49.1%)	
Lymph node metastasis				0.032*
NO	45	17 (37.8%)	28 (62.2%)	
YES	23	15 (65.2%)	8 (34.8%)	
TNM stage				0.169
I + II	46	19 (41.3%)	27 (58.7%)	
III + IV	22	13 (59.1%)	9 (40.9%)	

*P < 0.05

**Table 2:** Univariate and multivariate analyses of OS in ESCC patients

Characteristics	Univariate analysis				Multivariate analysis			
	B	*P*	HR	95%CI	B	*P*	HR	95%CI
Gender	−0.135	0.691	0.874	0.451–1.695	0.138	0.701	1.148	0.568–2.321
Age	−0.091	0.731	0.913	0.543–1.534	−0.303	0.303	0.738	0.414–1.316
Differentiation (Poor vs. well+moderate)	0.445	0.114	1.561	0.899–2.709	0.670	0.023*	1.955	1.099–3.478
pT stage (T1 + T2 vs. T3)	0.495	0.142	1.640	0.847–3.175	0.154	0.674	1.167	0.569–2.391
Lymph node metastasis	0.766	0.006*	2.151	1.246–3.715	0.769	0.009*	2.158	1.207–3.857
TNM stage (I + II vs. III + IV)	0.629	0.024*	1.875	1.087–3.235	0.150	0.747	1.162	0.467–2.891
Stromal FN content	0.771	0.004*	2.163	1.274–3.671	0.704	0.012 *	2.022	1.169–3.498

*P < 0.05

**Table 3:** Univariate and multivariate analyses of PFS in ESCC patients

Characteristics	Univariate analysis				Multivariate analysis			
	B	*P*	HR	95%CI	B	*P*	HR	95%CI
Gender	−0.439	0.228	0.645	0.316–1.315	−0.096	0.803	0.908	0.426–1.936
Age	−0.103	0.692	0.902	0.541–1.503	−0.390	0.178	0.677	0.384–1.194
Differentiation (poor vs. well+moderate)	0.237	0.384	1.267	0.743–2.160	0.283	0.326	1.327	0.755–2.333
pT stage (T1 + T2 vs. T3)	0.566	0.082	1.762	0.931–3.334	0.362	0.311	1.436	0.713–2.894
Lymph node metastasis	0.783	0.004*	2.188	1.281–3.736	0.578	0.039*	1.783	1.031–3.082
TNM stage (I + II vs. III + IV)	0.526	0.056	1.692	0.986–2.903	−0.204	0.661	0.816	0.328–2.027
Stromal FN content	1.034	0.000*	2.813	1.648–4.804	0.915	0.001*	2.498	1.444–4.319

*P < 0.05

### FN expression in ESCC cell lines and MSCs

**Fig. 2:**
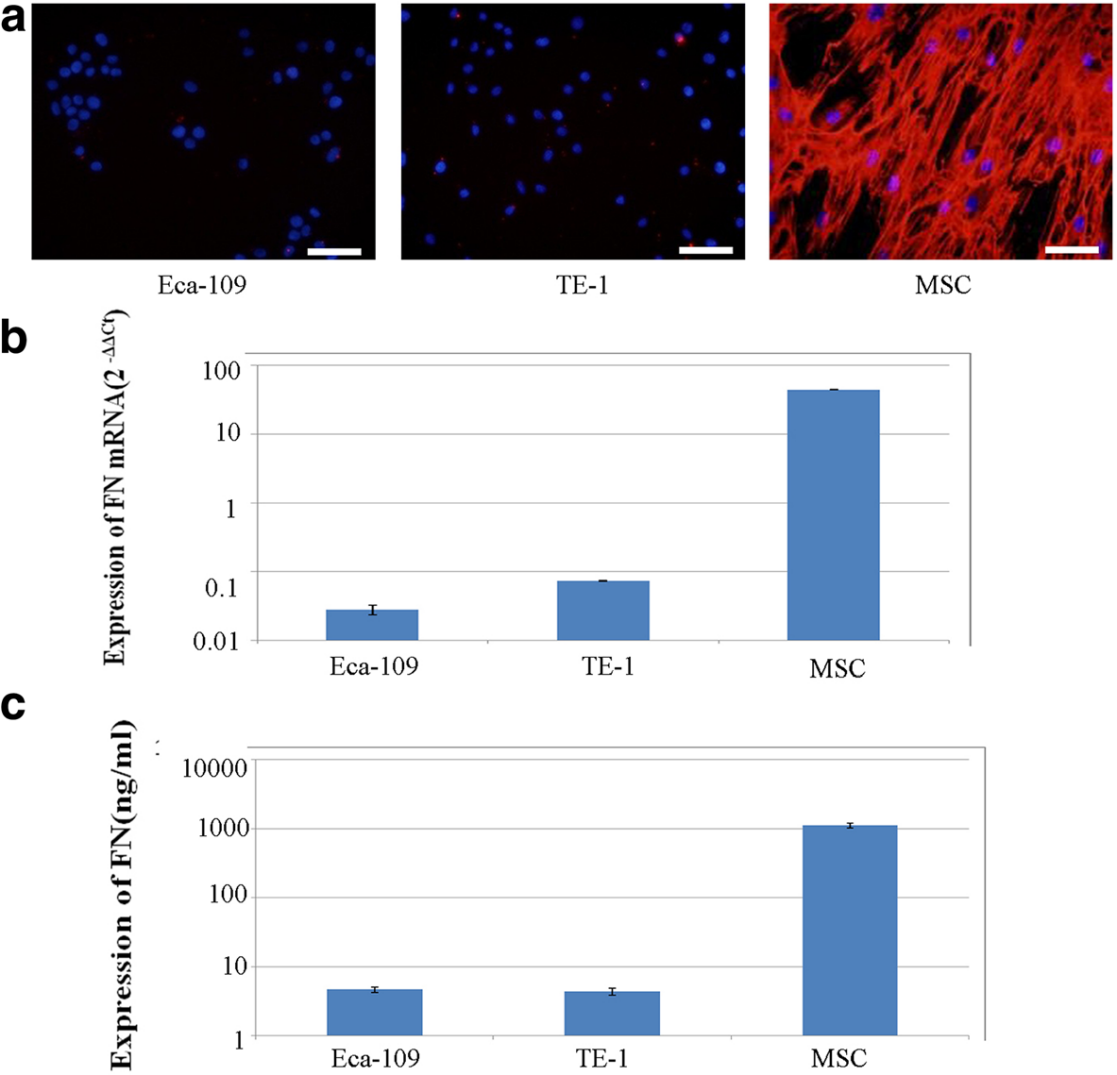
FN expression and secretion in the ESCC cell lines (Eca-109, TE-1) and MSCs. Immunofluorescence (**a**) (× 200) and RT-PCR (**b**) showed that FN protein and mRNA was expressed at low levels in ESCC cells and high levels of FN protein and mRNA in MSCs. **c** ELISAs showed high concentrations of FN in MSC-condition medium, demonstrating secretion of high levels of FN by MSCs. Scale bar = 100 μm

To investigate the expression of FN in ESCC cell lines and MSCs, we performed immunofluorescence and qRT-PCR analyses and ELISAs. These analyses showed that FN expression was low in ESCC cells, while MSCs expressed high levels of FN protein. Specifically, ELISAs showed that the average FN concentration in MSC-conditioned medium was 1147.0 ± 87.94 ng/ml; by comparison, the FN concentration in Eca-109 and TE-1 cell-conditioned medium was 4.5693 ± 0.423 and 4.813 ± 0.52 ng/ml, respectively (Fig. 2).

### Motility and migration ability of ESCC cells in a setting of high stromal FN content

**Fig. 3:**
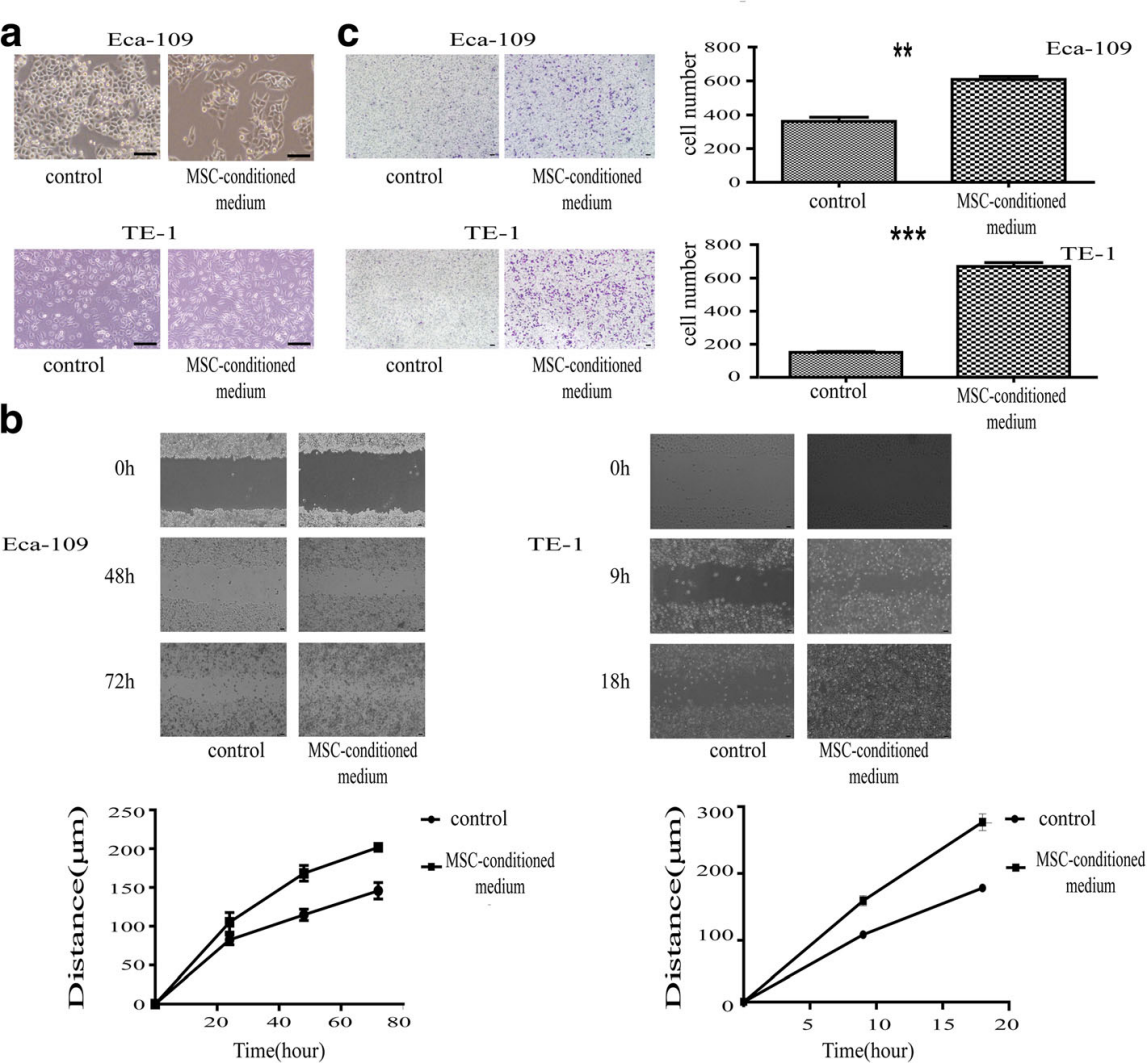
An in vitro environment simulating high stromal levels of FN improved the migration ability of the ESCC cell lines, Eca-109 and TE-1. **a** Changes in morphology induced by culture with MSC-conditioned medium for 42 h. Magnification, × 200. (**b, c**) Wound-healing assays (**b**) and Transwell assays (**c**) showed that culture with MSC-conditioned medium increased EECC cell migration ability. Magnification, × 40. The line chart in (**a**) shows the distance cells migrated over time, and the photographs in (**b**) show cells that traversed the membrane (Eca-109, *P* = 0.001; TE-1, *P* < 0.001). Scale bar = 100 μm

Because our data demonstrated that ESCC cells expressed low levels of FN, whereas the tumor stromal FN content was high, we speculated that stromal FN was secreted by mesenchymal cells and deposited in the stroma. To test this, we used MSCs as a model of stromal mesenchymal cells. To assess the motility and migration ability of ESCC cells in a setting of high stromal FN content, we cultured the ESCC cell lines, Eca-109 and TE-1, in the presence of MSC-conditioned medium (experimental group), mimicking a high stromal FN microenvironment, or DMEM (low-sugar) medium (control group). These experiments revealed that ESCC cells exhibited morphological changes when grown in MSC-conditioned medium, becoming longer and narrower (Fig. 3a). Wound-healing assay, used to detect changes in migration ability, showed that ESCC cells grown in a high stromal FN migrated more rapidly than cells in the control group (Fig. 3b). Similar results were obtained with Transwell assays, which showed that culture in the presence of high levels of FN enhanced the migration ability of ESCC cells (Fig. 3c).

## Discussion

In this study, we investigated stromal FN expression in ESCC specimens and ESCC cell lines, with the goal of elucidating the relationship between stromal FN expression and clinical/pathological features and prognosis of ESCC patients. We further cultured ESCC cell lines in a microenvironment containing a high FN content and assessed the effects of this high stromal FN-mimicking environment on the migration ability of ESCC cells—a key factor underlying tumor cell metastasis.

Numerous studies have reported the expression of FN in several types of carcinoma, demonstrating that the expression of FN in tumor tissue is higher than that in normal tissue [20–26]; however, there are notable differences between adenocarcinoma and squamous cell carcinoma. In adenocarcinoma, FN expressed in tumor cells [23, 24], whereas in squamous cell carcinoma, FN is deposited in the stroma, but is only expressed in a small percentage of tumor cells [25–27]. Although Sung et al. reported that FN expression in ESCC tumor tissuewas associated with the prognosis of ESCC patients, they did not report differences in FN expression between tumor cells and the tumor stroma [28]. In the current study, immunohistochemical results obtained from pathological sections of tumors from ESCC patients showed that FN was expressed in the stroma of ESCC tumors, but was detected in only a few tumor cells.

The relationship between stromal FN expression in ESCC specimens and clinical/pathological features and prognosis of patients has been a matter of controversy [6, 20–24]. In the current study, immunohistochemical detection of FN simultaneously in the stroma and tumor cells of clinical ESCC specimens showed that stromal FN expression was significantly associated with lymphatic metastasis (*P* = 0.032), but not with the age, gender, tumor stage or tumor-differentiation status of ESCC patients. Survival analyses further showed that ESCC patients with high FN expression in the tumor stroma had a shorter median OS (*P* = 0.002) and PFS (*P* < 0.001) compared with patients with low FN expression, with multivariate analyses showing that stromal FN expression was an independent prognostic factor.

In a previous study of oral squamous cell carcinomas, researchers speculated that FN was secrete by mesenchymal cells and deposited in the stroma, where it could impact the adhesion, escape, proliferation and differentiation of cells through paracrine effects on tumor cells [26]. However, a study of breast cancer suggested instead that FN was not only secreted by mesenchymal cells, such as cancer-associated fibroblasts (CAFs), but also by tumor cells themselves [29–32]. Thus, the source of FN in the tumor microenvironment is a matter of controversy, especially in the case of ESCC. In the current study, we measured FN expression in the ESCC cell lines, TE-1, Eca-109, at the mRNA and protein level using qRT-PCR and immunofluorescence, respectively, showing that these ESCC cell lines expressed only low levels of FN. Consistent with this, ELISAs showed that FN secretion by ESCC cell lines was also low. Accordingly, we speculate that most FN in the ESCC tumor stroma is secreted by mesenchymal cells and deposited in the stroma.

In most solid tumors, the most-mature mesenchymal cell is the mechanocyte, and one of the source of mechanocytes is the MSC [33]. Using qRT-PCR, immunofluorescence and ELISA, we confirmed that MSCs highly expressed and secreted FN, a finding that is accord with the speculation that mesenchymal cells highly express FN. Based on recent studies that have used MSCs to simulate stromal cells in the tumor microenvironment in vivo [34], we created an in vitro model in which the ESCC cell lines, TE-1 and Eca-109, were cultured in MSC-conditioned medium containing high levels of FN, mimicking the effect of mesenchymal cells in the tumor microenvironment. Subsequent microscopic analyses and wound-healing and Transwell assays showed that culture of ESCC cells in an environment containing high levels of FN altered the morphology of these cells, yielding long, spindle-shaped cells with increased motility and migration ability. Therefore, we conclude that an environment containing high levels of FN enhances the motility and migration ability of ESCC cells, suggesting that FN secreted by mesenchymal cells plays an important role in these ESCC cell properties.

FN exerts its effects through signaling pathways initiated by binding to cellular receptors called integrins [35–37]. Some studies have reported that FN stimulates human cancer cell growth through binding to α5β1 integrin receptors on the cell surface and activation of the MEK1/ERK pathway [38–41]. Similar studies have confirmed that FN activates the ERK pathway in ESCC, and shown that activation of this pathway is associated with tumor development [42]. Additional in vitro studies have shown that MEK1/ERK signaling can be inhibited by blocking fibronectin-a5b1 interactions, depending upon the reagents used and the cells studied [43–46]. Such studies pave the way for the development of targeted drugs for the treatment of ESCC.

## Conclusion

A high stromal-FN environment promotes the motility and migration ability of ESCC cells. Importantly, elevated stromal FN is closely associated with poor prognosis of ESCC patients, and has significance for guiding clinical diagnosis and predicting prognosis.

## Abbreviations

CI: Confidence interval
ESCC: Esophageal squamous cell carcinoma
FN: Fibronectin
HR: Hazards ratio
MSC: Mesenchymal stem cell
OS: Overall survival
PFS: Progression-free survival
pN: Pathologic lymph node stage
pT: Pathologic tumor stage

## References

[1] Pennathur A, Gibson MK, Jobe BA, Luketich JD (2013). Oesophageal carcinoma. Lancet.

[2] Siegel RL, Miller KD, Jemal A (2017). Cancer statistics, 2017. CA Cancer J Clin.

[3] Torre LA, Bray F, Siegel RL, Ferlay J, Lortet-Tieulent J, Jemal A (2015). Global Cancer statistics, 2012. CA Cancer J Clin.

[4] Singh P, Carraher C, Schwarzbauer JE (2010). Assembly of fibronectin extracellular matrix. Annu Rev Cell Dev Biol.

[5] Gould VE, Kouloulis GK, Virtanen I (1990). Extracellular matrix proteins and their receptors in the normal, hyperplastic and neoplastic breast. Cell Diff Dev.

[6] Ioachim E, Charchanti A, Briasoulis E, Karavasilis V, Tsanou H, Arvanitis DL, Agnantis NJ, Pavlidis N (2002). Immunohistochemical expression of extracellular matrix components tenascin, fibronectin, collagen type IV and laminin in breast cancer: their prognostic value and role in tumour invasion and progression. Eur J Cancer.

[7] Han JY, Kim HS, Lee SH, Park WS, Lee JY, Yoo NJ (2003). Immunohistochemical expression of integrins and extracellular matrix proteins in non-small cell lung cancer: correlation with lymph node metastasis. Lung Cancer.

[8] Kamoshida G, Matsuda A, Miura R, Takashima Y, Katsura A, Tsuji T (2013). Potentiation of tumor cell invasion by co-culture with monocytes accompanying enhanced production of matrix metalloproteinase and fibronectin. Clin Exp Metastasis.

[9] Jia D, Yan M, Wang X, Hao X, Liang L, Liu L, Kong H, He X, Li J, Yao M (2010). Development of a highly metastatic model that reveals a crucial role of fibronectin in lung cancer cell migration and invasion. BMC Cancer.

[10] Núñez MA, de Matos FR, Freitas Rde A, Galvão HC (2013). Immunohistochemical study of integrin α_5_ β_1_, fibronectin, and Bcl-2 in normal oral mucosa, inflammatory fibroepithelial hyperplasia, oral epithelial dysplasia, and oral squamous cell carcinoma. Appl Immunohistochem Mol Morphol.

[11] Lee JM, Dedhar S, Kalluri R, Thompson EW (2006). The epithelial–mesenchymal transition: new insights in signaling, development,and disease. J Cell Biol.

[12] Sudo T, Iwaya T, Nishida N, Sawada G, Takahashi Y, Ishibashi M, Shibata K, Fujita H, Shirouzu K, Mori M, Mimori K (2013). Expression of mesenchymal markers vimentin and fibronectin: the clinical significance in esophageal squamous cell carcinoma. Ann Surg Oncol.

[13] Zhang X, Huang W, Chen X, Lian Y, Wang J, Cai C, Huang L, Wang T, Ren J, Xiang AP (2017). CXCR5-Overexpressing Mesenchymal Stromal Cells Exhibit Enhanced Homing and Can Decrease Contact Hypersensitivity. Mol Ther.

[14] Peng Y, Chen X, Liu Q, Xu D, Zheng H, Liu L, Liu Q, Liu M, Fan Z, Sun J, Li X, Zou R, Xiang AP (2014). Alteration of naïve and memory B-cell subset in chronic graft-versus-host disease patients after treatment with mesenchymal stromal cells. Stem Cells Transl Med.

[15] Liu X, Wu M, Peng Y, Chen X, Sun J, Huang F, Fan Z, Zhou H, Wu X, Yu G, Zhang X, Li Y, Xiao Y, Song C, Xiang AP, Liu Q (2014). Improvement in poor graft function after allogeneic hematopoietic stem cell transplantation upon administration of mesenchymal stem cells from third-party donors: a pilot prospective study. Cell Transplant.

[16] Qin S, Zhang B, Xiao G, Sun X, Li G, Huang G, Gao X, Li X, Wang H, Yang C, Ren H (2016). Fibronectin protects lung cancer cells against docetaxel-induced apoptosis by promoting Src and caspase-8 phosphorylation. Tumour Biol.

[17] Yu ST, Zhong Q, Chen RH, Han P, Li SB, Zhang H, Yuan L, Xia TL, Zeng MS, Huang XM (2018). CRLF1 promotes malignant phenotypes of papillary thyroid carcinoma by activating the MAPK/ERK and PI3K/AKT pathways. Cell Death Dis.

[18] He XT, Li X, Yin Y, Wu RX, Xu XY, Chen FM (2018). The effects of conditioned media generated by polarized macrophages on the cellular behaviours of bone marrow mesenchymal stem cells. J Cell Mol Med.

[19] Nutter F, Holen I, Brown HK, Cross SS, Evans CA, Walker M, Coleman RE, Westbrook JA, Selby PJ, Brown JE, Ottewell PD (2014). Different molecular profiles are associated with breast cancer cell homing compared with colonisation of bone: evidence using a novel bone-seeking cell line. Endocr Relat Cancer.

[20] Steffens S, Schrader AJ, Vetter G, Eggers H, Blasig H, Becker J, Kuczyk MA, Serth J (2012). Fibronectin 1 protein expression in clear cell renal cell carcinoma. Oncol Lett.

[21] Richter P, Junker K, Franz M, Berndt A, Geyer C, Gajda M, Kosmehl H, Berndt A, Wunderlich H (2008). IIICS de novo glycosylated fibronectin as a marker for invasiveness in urothelial carcinoma of the urinary bladder (UBC). J Cancer Res Clin Oncol.

[22] Jha RK, Ma Q, Chen S, Sha H, Ding S (2009). Relationship of fibronectin and CD44v6 expression with invasive growth and metastasis of liver cancer. Cancer Investig.

[23] Yun JA, Kim SH, Hong HK, Yun SH, Kim HC, Chun HK, Cho YB, Lee WY (2014). Loss of E-cadherin expression is associated with a poor prognosis in stage III colorectal cancer. Oncology.

[24] Bae YK, Kim A, Kim MK, Choi JE, Kang SH, Lee SJ (2013). Fibronectin expression in carcinoma cells correlates with tumor aggressiveness and poor clinical outcome in patients with invasive breast cancer. Hum Pathol.

[25] Mhawech P, Dulguerov P, Assaly M, Ares C, Allal AS (2005). EB-D fibronectin expression in squamous cell carcinoma of the head and neck. Oral Oncol.

[26] Lyons AJ, Bateman AC, Spedding A, Primrose JN, Mandel U (2001). Oncofetal fibronectin and oral squamous cell carcinoma. Br J Oral Maxillofac Surg.

[27] Kosmehl H, Berndt A, Strassburger S, Borsi L, Rousselle P, Mandel U, Hyckel P, Zardi L, Katenkamp D (1999). Distribution of laminin and fibronectin isoforms in oral mucosa and oral squamous cell carcinoma. Br J Cancer.

[28] Sung CO, Park CK, Kim SH (2011). Classification of epithelial–mesenchymal transition phenotypes in esophageal squamous cell carcinoma is strongly associated with patient prognosis. Mod Pathol.

[29] Fernandez-Garcia B, Eiró N, Marín L, González-Reyes S, González LO, Lamelas ML, Vizoso FJ (2014). Expression and prognostic significance of fibronectin and matrix metalloproteases in breast cancer metastasis. Histopathology.

[30] Cunha GR, Hayward SW, Wang YZ, Ricke WA (2003). Role of the stromal microenvironment in carcinogenesis of the prostate. Int J Cancer.

[31] Tlsty TD, Hein PW (2001). Know thy neighbor: stromal cells can contribute oncogenic signals. Curr Opin Genet Dev.

[32] Hu M, Carles-Kinch KL, Zelinski DP, Kinch MS (2004). EphA2 induction of fibronectin creates a permissive microenvironment for malignant cells. Mol Cancer Res.

[33] Spaeth EL, Dembinski JL, Sasser AK, Watson K, Klopp A, Hall B, Andreeff M, Marini F (2009). Mesenchymal stem cell transition to tumor-associated fibroblasts contributes to fibrovascular network expansion and tumor progression. PLoS One.

[34] Martin FT, Dwyer RM, Kelly J, Khan S, Murphy JM, Curran C, Miller N, Hennessy E, Dockery P, Barry FP, O'Brien T, Kerin MJ (2010). Potential role of mesenchymal stem cells (MSCs) in the breast tumour microenvironment: stimulation of epithelial to mesenchymal transition (EMT). Breast Cancer Res Treat.

[35] Miyamoto S, Akiyama SK, Yamada KM (1995). Synergistic roles for receptor occupancy and aggregationin integrin transmembrane function. Science.

[36] Roman J (1996). Extracellular matrix and lung inflammation. Immunol Res.

[37] Akiyama SK, Olden K, Yamada KM (1995). Fibronectin and integrins in invasion and metastasis. Cancer Metastasis Rev.

[38] Han S, Sidell N, Roser-Page S, Roman J (2004). Fibronectin stimulates human lung carcinoma cell growth by inducing cyclooxygenase-2 (COX-2) expression. Int J Cancer.

[39] Aguirre Ghiso JA, Kovalski K, Ossowski L (1999). Tumor dormancy induced by downregulation of urokinase receptor in human carcinoma involves integrin and MAPK signaling. J Cell Biol.

[40] Malecki M, Trembacz H, Sroczynska P, Janik P (2000). Signal transduction in adherent and non-adherent human cell lines after fibronectin stimulation. Oncol Rep.

[41] Ritzenthaler JD, Han S, Roman J (2008). Stimulation of lung carcinoma cell growth by fibronectin–integrin signaling. Mol BioSyst.

[42] Zhang J, Zhi H, Zhou C, Ding F, Luo A, Zhang X, Sun Y, Wang X, Wu M, Liu Z (2005). Up-regulation of fibronectin in oesophageal squamous cell carcinoma is associated with activation of the Erk pathway. J Pathol.

[43] Han S, Rivera HN, Roman J (2005). Peroxisome proliferator-activated receptor-gamma ligands inhibit alpha5 integrin gene transcription in non-small cell lung carcinoma cells. Am J Respir Cell Mol Biol.

[44] Wilson SH, Ljubimov AV, Morla AO, Caballero S, Shaw LC, Spoerri PE, Tarnuzzer RW, Grant MB (2003). Fibronectin fragments promote human retinal endothelial cell adhesion and proliferation and ERK activation through alpha5beta1 integrin and PI 3-kinase. Invest Ophthalmol Vis Sci.

[45] Kapur R, Cooper R, Zhang L, Williams DA (2001). Cross-talk between alpha-(4)beta(1)/alpha(5)beta(1) and c-kit results in opposing effect on growth and survival of hematopoietic cells via the activation of focal adhesion kinase, mitogen-activated protein kinase, and Akt signaling pathways. Blood.

[46] Liang F, Atakilit A, Gardner DG (2000). Integrin dependence of brain natriuretic peptide gene promoter activation by mechanical strain. J Biol Chem.

